# The Multidrug Resistance-Reversing Activity of a Novel Antimicrobial Peptide

**DOI:** 10.3390/cancers12071963

**Published:** 2020-07-19

**Authors:** Qiu-Xu Teng, Xiaofang Luo, Zi-Ning Lei, Jing-Quan Wang, John Wurpel, Zuodong Qin, Dong-Hua Yang

**Affiliations:** 1 Department of Pharmaceutical Sciences, College of Pharmacy and Health Sciences, St. John’s University, Queens, NY 11439, USA; qiuxu.teng15@stjohns.edu (Q.-X.T.); zining.lei14@stjohns.edu (Z.-N.L.); Jingquan.wang16@stjohns.edu (J.-Q.W.); wurpelj@stjohns.edu (J.W.); 2 Research Center of Biochemical Engineering Technology, College of Chemistry and Bioengineering, Hunan University of Science and Engineering, Yongzhou 425199, China; luoxf@huse.edu.cn

**Keywords:** antimicrobial peptide, multidrug resistance, reversal agents, ABC transporter, combination therapy

## Abstract

The overexpression of ATP-binding cassette (ABC) transporters is a common cause of multidrug resistance (MDR) in cancers. The intracellular drug concentration of cancer cells can be decreased relative to their normal cell counterparts due to increased expression of ABC transporters acting as efflux pumps of anticancer drugs. Over the past decades, antimicrobial peptides have been investigated as a new generation of anticancer drugs and some of them were reported to have interactions with ABC transporters. In this article, we investigated several novel antimicrobial peptides to see if they could sensitize ABCB1-overexpressing cells to the anticancer drugs paclitaxel and doxorubicin, which are transported by ABCB1. It was found that peptide XH-14C increased the intracellular accumulation of ABCB1 substrate paclitaxel, which demonstrated that XH-14C could reverse ABCB1-mediated MDR. Furthermore, XH-14C could stimulate the ATPase activity of ABCB1 and the molecular dynamic simulation revealed a stable binding pose of XH-14C-ABCB1 complex. There was no change on the expression level or the location of ABCB1 transporter with the treatment of XH-14C. Our results suggest that XH-14C in combination with conventional anticancer agents could be used as a novel strategy for cancer treatment.

## 1. Introduction

The incidence of cancer has increased as a result of growing population, aging, and other risk factors [1,2]. After decades of investigation and development, chemotherapies started to reduce the cancer incidence and cancer death rate. Despite that, the bright future of chemotherapies were gradually shadowed because of endogenous multidrug resistance (MDR) or MDR acquisition due to chemotherapies [3], leading to refractory cancers and tumor recurrences, which ultimately contribute to increasing cancer-related deaths. As MDR refers to the resistance of cancer cells to various different structural and functional chemotherapeutic drugs [4], it is a major factor of failure of cancer chemotherapy [5]. The mechanisms of MDR could be classified into several categories, with the major one being the enhancement of drug efflux by transporters on cancer cell membranes [6].

The ATP-binding cassette (ABC) transporter family is a protein superfamily with 49 different members categorized by gene sequence and structural similarities [3]. They are the main cell membrane transporters and are organized into seven subfamilies [7]. Many human ABC proteins, including P-glycoprotein (P-gp/ABCB1/MDR1), breast cancer resistance protein (BCRP/ABCG2/ABCP/MXR), and multidrug resistance protein 1 (MRP1/ABCC1) are efflux transporters [8], and have been recognized as the culprit in the development of MDR. These membrane transporters have the ability to enhance the outflow of chemotherapeutic drugs to reduce the intracellular accumulation of drugs, which is one of the most common causes of MDR [9,10]. Human ABCB1 transporter was the first recognized ABC transporter of which its overexpression could induce drug resistance of cancer cells to a series chemotherapeutic drugs like paclitaxel, doxorubicin, and vincristine [11].

ABCB1 is a 170 kDa membrane transporter that is ubiquitously expressed in kidney, intestine, brain, and placenta [11]. Current strategies to overcome MDR mainly focus on the development of reversal agents that can inactivate or inhibit the efflux function of ABC transporters so that the intracellular concentration of anticancer drugs could be enhanced [12,13,14,15,16,17]. Clinical applications of the combination of an ABCB1 modulator and an anticancer drug have been investigated as a possible strategy to overcome ABCB1-mediated drug efflux for a long time [18,19]. Three generations of ABCB1 inhibitors and other compounds have been developed over the past few decades. Verapamil, a calcium channel blocker, is one of the first generation MDR reversal agents [18]. However, the in vivo effective concentration for reversal is too high to achieve safely, and the dose of verapamil required is much higher than clinically relevant dose, carrying the risk of toxicity in almost all patients [20]. This led to its limited clinical application as a reversal agent. The second generation of ABCB1 inhibitors were synthesized around the first generation pharmacophores to increase the affinity to ABCB1 while reducing dose-limiting toxicity, and the third generation was specifically designed to have high affinity for ABCB1 and low pharmacokinetic interactions [18,19].However, few developed inhibitors have been approved for use in the market because of lacking significant clinical efficacy, or concerns about their clinical safety [19]. Thus, investigating and establishing effective and nontoxicABCB1 inhibitors to reverse MDR in cancers has become a pressing need.

Due to the rapid growth of cancer cases worldwide and the increase in chemotherapy resistance, there is an inevitable demand for the development and screening of potential reversal agents that may be effective against MDR. In the past few decades, although challenging, scientists have focused on the development of new compounds as resistance reversal agents. Recently, it has been shown that some of the tyrosine kinase inhibitors (TKIs) could be used as MDR reversal agents in combination with conventional anticancer drugs [21], which could inhibit the efflux of anticancer drugs in drug-resistant cancer cells with overexpression of ABC transporters [9,10]. Most of the TKIs act as competitive or noncompetitive inhibitors of the ABC transporters. ABC transporters are not only expressed in cancer cells but also in normal tissues including the liver, kidneys, gastrointestinal tract, and most importantly, the blood-brain barrier [22,23,24]. As TKIs are not specific to ABC transporters in cancer cells, they may also cause cytotoxicity to other normal tissue. In addition, point mutations in the kinase region can lower sensitivity of kinases to TKIs, andare one of the mechanisms of resistance to TKIs [25,26]. Besides the mutations in the TKS domain, there are other unknown mechanisms that may contribute to TKIs resistance [27].

Peptides have a number of beneficial characteristics including low adverse toxic effects and possibility of reducing cancer cell drug resistance [28,29,30] because their targeted position is the cell membrane, which enhances their value to meet the need of new therapies compared to chemically synthesized agents [31]. According to recent reports, some antimicrobial peptides exhibited anticancer effect on cancer MDR cells with less toxicity [32,33], suggesting that they could be therapeutic candidates for new anticancer drugs [34,35]. Antimicrobial peptides are a kind of defense substance produced spontaneously by microorganisms that can kill bacteria that are competing for nutrients [28]. Although they have diverse sequences, antimicrobial peptides also share some fundamental structural features including a short size, carrying a positive net charge, and feature membrane permeability or an amphiphilic nature which allows insertion into the cell membrane [29]. Due to the hydrophobic and negatively charged environments of cancer cell membranes, the hydrophobic and cationic nature of antimicrobial peptides could facilitate preferential binding to cancer cells but not normal cells are zwitterionic [30,36].In addition to binding to the cell membrane which results in cell death, antimicrobial peptides can also block the interaction between growth factors and their receptors, activating anti-angiogenic effects; inhibiting specific kinase/protease which promote the growth, invasion, and metastasis of tumor; and inhibiting special functional proteins to halt the progression of cancer [34,35]. These different mechanisms of cancer cell death may overcome the limitations of traditional chemotherapeutic agents.

We have designed three antimicrobial peptides XH-14A (FIKRIARLLRKIKR), XH-14B (FIKRIARLLRKIFR), and XH-14C (FIKRIARLLRKIWR) based on the template of BmKn2 (FIGAIARLLSKIF), an antibacterial peptide with 13 amino acids identified from ButhusmartensiiKasch [35]. These peptides displayed potency against both Gram-negative and Gram-positive bacteria. The replacements of one or more amino acid residues by other amino acid residues were involved in the design and modification of the antimicrobial peptides. As some antimicrobial peptides have anticancer activities and do not lead to drug resistance in cancer cells, the anticancer activity of these peptides was tested. The possible reversal effect of these novel peptides and whether the reversal activity is associated with the overexpression of ABCB1 transporter was the focus of this project. Our findings suggested that peptide XH-14C has potential ABCB1-overexpressing MDR reversing activity which would allow further design or modification of these peptides.

## 2. Results

### 2.1. Cytotoxicity of Three Peptides on ABC Transporter-Overexpressing Cell Lines

**Figure 1:**
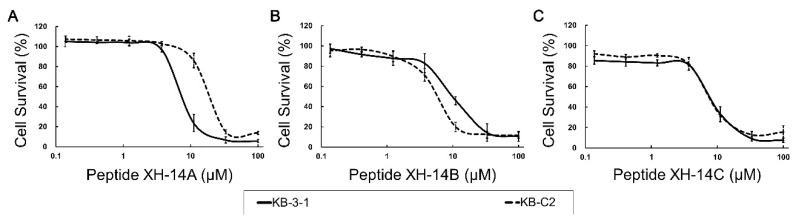
Dose-response curve of **A**) XH-14A, **B**) XH-14B and **C**) XH-14C, on drug-selected ABCB1-overexpressing cell lines (KB-C2) and its parental drug-sensitive cell line (KB-3-1) with gradient concentrations. Each point with error bar represents the mean ± SD of the cytotoxicity with different concentrations calculated from at least three independent triplicate experiments.

The cytotoxicity of the antimicrobial peptides XH-14A, XH-14B, and XH-14C were analyzed by MTT colorimetric assay. From the dose-response curve in Figure 1, the IC_50_ values of peptide XH-14A, XH-14B, and XH-14C in ABCB1-overexpressing cancer cells (KB-C2) were 22.45 µM, 8.38 µM, and 7.82 µM, respectively. Meanwhile, the IC_50_ values of the peptides in parental cells (KB-3-1) were determined to be 8.73 µM, 10.02 µM, and 8.12 µM, respectively. The IC_50_ values in ABCG2-overexpressing cells were 8.22 µM, 7.28 µM, and 8.30 µM in NCI-H460/MX20 cells and in their corresponding parental cells NCI-H460 were 10.14 µM, 9.37 µM, and 9.07 µM, respectively (Figure S1). The concentration of these peptides at 3 µM, a concentration that would not produce significant cytotoxicity (at least 85% cell survival) was selected for further study.

### 2.2. Cytotoxicity of ABC Substrates in Combination with the Peptide

To test the reversal effect of these three peptides in ABC transporter-mediated MDR, drug-selected MDR human cancer cell lines, KB-C2 and NCI-H460/MX20, and their parental cell lines, KB-3-1 and NCI-H460, were used to examine the ABCB1 substrates paclitaxel and doxorubicin and ABCG2 substrates mitoxantrone and topotecan. The drug resistance profile in Table 1 and Table S1 demonstrated that only peptide XH-14C significantly potentiated the cytotoxicity of paclitaxel and doxorubicin in ABCB1-overexpressing KB-C2, but none of the three peptides demonstrated reversal effects on ABCG2-overexpressing NCI-H460/MX20 cells. As the MDR in drug-selected cancer cells might be multifactorial, the transfected ABCB1-overexpressing cell line (HEK293/ABCB1) was used to verify the actual role of ABCB1 and similar reversal effects were observed (Table 2). In addition, no significant change in the drug resistant profile of cisplatin, which is not a substrate of ABCB1 transporter, in both drug-selected and transfected ABCB1-overexpression cells was observed with or without the peptide. These results indicated that peptide XH-14C could reverse MDR of cancer cells mediated by ABCB1-overexpression but not ABCG2-overexpression.

### 2.3. XH-14C on ATPase Activity of ABCB1 and ABCG2 Transporters

**Figure 2:**
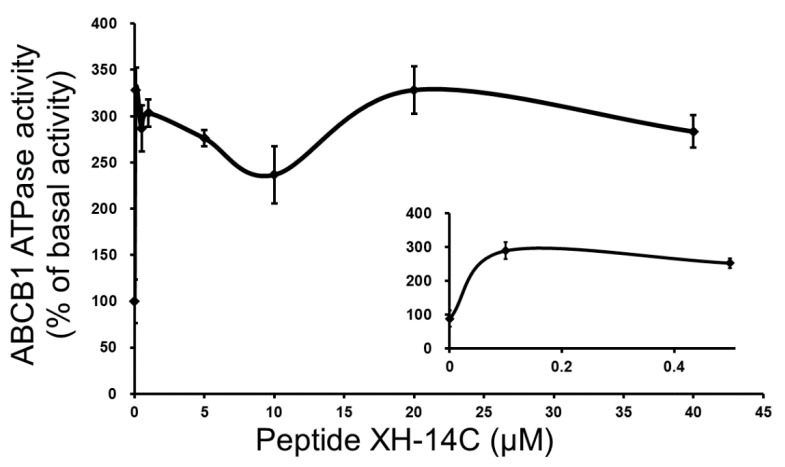
The vanadate sensitive ABCB1 transporter specific ATPase activity was stimulated by XH-14C.Gradient concentration of XH-14C (0–40 μM) as x-axis and ABCB1 ATPase activity represented in percentage of basal activity as y-axis. The small inner figure shows the lower concentrations (0–0.5 μM) of XH-14C versus ATPase activity. The mean ± SD is demonstrated as the points with error bars calculated based on three independent experiments.

As the ABC transporters hydrolyze ATP to facilitate the efflux of anticancer drugs, the possible mechanism behind the reversal effect of XH-14C was investigated by analyzing the ATPase activity of ABCB1 and ABCG2 in the presence of XH-14C at various concentrations. Figure 2 demonstrated that XH-14C stimulated the ATPase activity of ABCB1 but had almost no effect on the ATPase activity of ABCG2 (Figure S2). A maximal stimulation of 3.28-fold on ABCB1 ATPase activity and a maximal stimulation of 1.03-fold on ABCG-2 ATPase activity were observed. In addition, in a concentration-dependent manner, a concentration of 0.05 μM, which is much lower than the reversal effect concentration in the reversal assay, was calculated as the essential concentration for half simulation of ABCB1 ATPase activity. These results suggested that XH-14C may reverse ABCB1-mediated MDR by affecting the ATPase activity through interaction with ABCB1 protein.

### 2.4. Interaction of XH-14C with ABCB1

**Figure 3:**
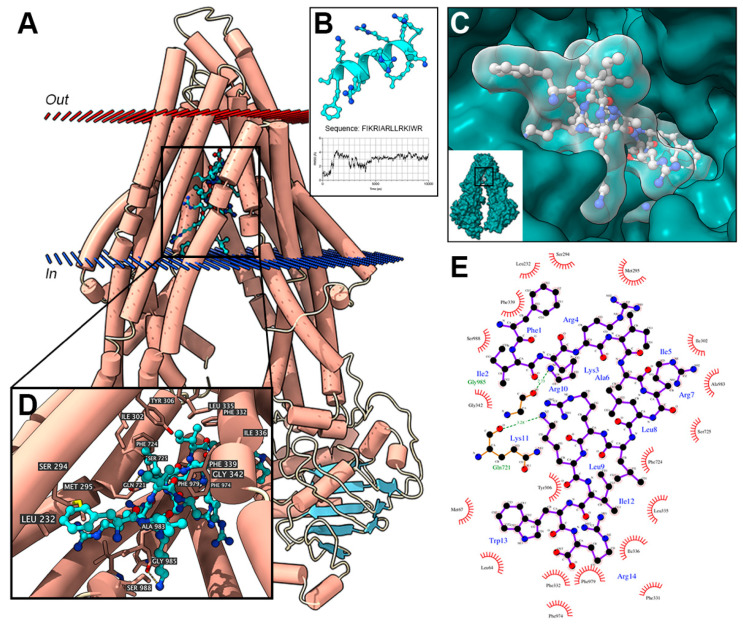
Docking simulation of XH-14C with ABCB1. (**A**). Best scoring poses of XH-14C in the drug binding pocket of ABCB1. XH-14C is depicted with colored sticks. Cyan: carbon; red: oxygen, blue: nitrogen. ABCB1 (4M2T) is depicted with colored tubes and ribbons. Orange: helices; light blue: strands; white: coils. Cytoplasmic membranes are depicted with red and blue planes. Red: periplasmic membrane; blue: cytoplasmic membrane. (**B**). Conformation of the best scoring XH-14C and results of molecular dynamics simulation with root mean square deviation (Å) on y-axis and time (ps) on x-axis.(**C**). Geometric poses of XH-14C docked into ABCB1 binding pocket. Protein is displayed with solid green surface. XH-14C is displayed with transparent white solid surface. XH-14H molecules are colored by heteroatoms: Grey, carbon; blue, nitrogen; red, oxygen. (**D**). Details of the interaction between XH-14C and ABCB1 binding pocket as involved residues are displayed with colored sticks with labels and hydrogen bonds depicted with yellow dashed lines. (**E**). 2D view of the interaction between XH-14C and ABCB1 binding pocket. XH-14C is depicted with balls and sticks. Black, carbon; blue: nitrogen; red, oxygen. Residues forming hydrophobic interactions are depicted with red circles with labels. Hydrogen bonds are displayed with bond length as green dash lines.

To study the potential interaction between XH-14C and ABCB1 binding pocket, a docking analysis was performed. Figure 3 demonstrated that XH-14C binds tightly in the ABCB1 binding pocket and stabilized by hydrogen bonds and hydrophobic interactions. Specifically, the lysine of XH-14C was stabilized by a hydrogen bond with Gln721 of ABCB1. Also, the arginine of XH-14C was stabilized by a hydrogen bond with Gly985 of ABCB1. These hydrogen bonds provide essential forces to the binding affinity of XH-14C and ABCB1. Additionally, XH-14C was also buried in tight hydrophobic cavities formed by residues including Phe724, Leu335, Tyr306, Gly342, Phe339 and Leu232.

### 2.5. Effects of XH-14C on ABCB1 Transporter’s Function

**Figure 4:**
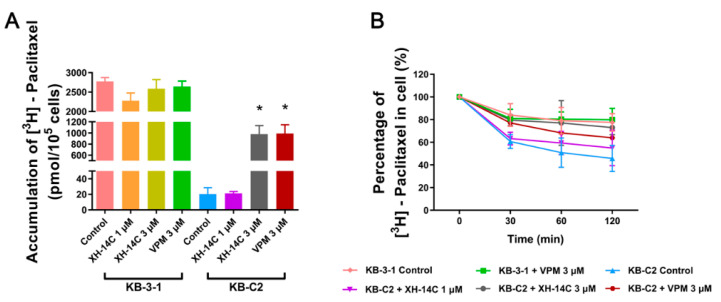
The drug accumulation and efflux activity of XH-14C. (**A**) The intracellular level of [^3^H]-paclitaxel in KB-3-1 and KB-C2 cells. Columns with error bar represent mean ± SD calculated from three independent duplicate experiments. * represents *p <* 0.05, compared with the KB-C2 control group. VPM, verapamil. (**B**) The efflux of [^3^H]-paclitaxel measured and calculated as the percentage of intracellular concentrations of [^3^H]-paclitaxel at different time points in KB-3-1 and KB-C2 cells. The points with error bars are calculated and represent the mean ± SD from three independent duplicate experiments. VPM, verapamil.

The ABCB1 transporter functions as the drug pump on the cancer cell membrane to efflux the anticancer drugs out of cancer cells, therefore bringing down the intracellular level of anticancer drugs. The effects of XH-14C on accumulation and efflux of ABCB1 substrate paclitaxel were determined by [^3^H]-paclitaxel measurement in KB-3-1 and ABCB1-overexpressing KB-C2 cells. The intracellular [^3^H]-paclitaxel with anticancer drug alone in KB-C2 cells was more than 100-fold lower than that in KB-3-1 cells after 2 h incubation with XH-14C (Figure 4A). When compared with [^3^H]-paclitaxel alone, the combination with 3 µM XH-14C could notably improve the intracellular accumulation of [^3^H]-paclitaxel about 50-folds in KB-C2 cells. This result was comparable to the combination with 3 μM verapamil, a known inhibitor of ABCB1. The efflux of [^3^H]-paclitaxel observed in Figure 4B demonstrated that the efflux function of ABCB1 on KB-C2 cells was dramatically inhibited with the combination of 3 µM XH-14C as the concentration of [^3^H]-paclitaxel in KB-C2 cells was similar to that in KB-3-1 cells after 2 h, while the drug intracellular concentration of KB-C2 cells decreased by about 50% without XH-14C. The cellular concentration of [^3^H]-paclitaxel did not significantly change after 2 h treatment either with or without a reversal agent in KB-3-1 cells.

### 2.6. Effect of XH-14C on the expression of ABCB1

**Figure 5:**
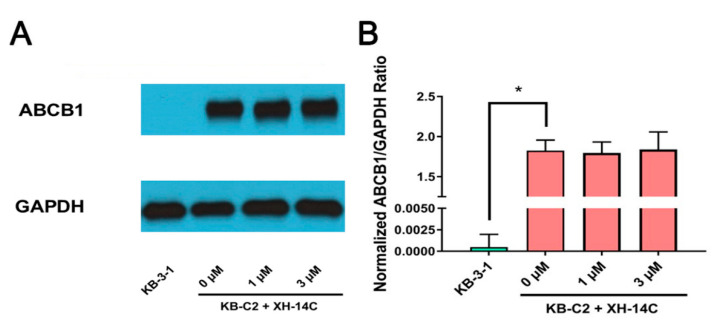
Western blotting of XH-14C to quantify ABCB1 expression level. (**A**) ABCB1 protein expression after incubation with different concentrations of XH-14C for 72 h in KB-C2 cells. KB-3-1 cells without any treatment used as the negative control of ABCB1 protein expression. GAPDH (glyceraldehyde 3-phosphate dehydrogenase) was used as loading control. (**B**) The relative intensity of the expression of ABCB1 compared with the expression of GAPDH. Expression level quantification by gray scale values calculated by ImageJ software (NIH, Bethesda, MD, USA). *, represents *p* < 0.05.

Western blotting was used to analyze and quantify the protein expression level of ABCB1. To normalize and calculate the expression level, GAPDH protein (37 kDa) was used as a loading control and the relative expression level of ABCB1 was calculated as the measurement of ABCB1 expression divided by the measurement of GAPDH expression. As shown in Figure 5, compared to KB-3-1 cells, KB-C2 had dramatic overexpression of ABCB1 protein (172 kDa) while different concentrations (0, 1, 3 μM) of XH-14C treatment at 72 h on KB-C2 cells all had similar relative expression levels of ABCB1.

### 2.7. Effects of XH-14C on the Subcellular Localization of ABCB1

**Figure 6:**
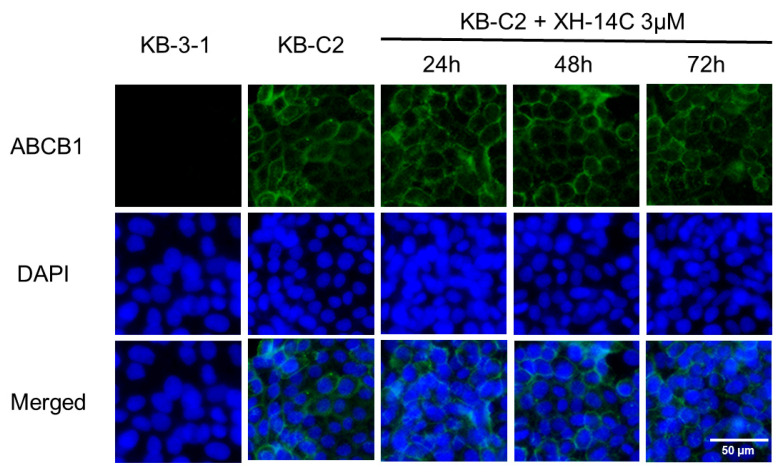
Immunofluorescence pictures of KB-3-1 and KB-C2 cells.ABCB1 protein expression detected by immunofluorescence after incubation with 3 μM XH-14C for different time periods. DAPI (4′,6-diamidino-2-phenylindole) was used to localize the cells by counterstaining the nuclei. Pictures have been modified by Photoshop software for merged comparison. Scale bar: 50 μM

Immunofluorescent staining was used to quantify the expression of ABCB1 and the subcellular localization of ABCB1 on cell membranes. Figure 6 indicates that ABCB1 transporter was overexpressed on the cell membrane of KB-C2 cells but not on the membrane of KB-3-1 cells. With the same concentration of 3 μM XH-14C and different treatment time periods (24, 48, and 72 h), no remarkable alternation of expression level or subcellular distribution of ABCB1were observed in KB-C2 cells.

## 3. Discussion

The efflux of anticancer drugs constitutes the majority of MDR, which is a known molecular mechanism of chemotherapeutic failure. Therefore, development of novel reversal agents that could inhibit the efflux functions of ABC transporters is an urgent need to advance chemotherapy. ABCB1 is a typical transporter of the ABC protein superfamily. So far, there are a number of studies focused on the investigation of reversal agents for ABCB1 [37,38]. However, the potent reversal agents investigated in vitro did not show similar effectsin vivo; in addition, they had the problems of bioavailability, neurotoxicity, and synergetic effects.

Antimicrobial peptides are novel anticancer drugs with less possibility of causing drug resistance. Some primary differences between the cell membranes of human tumor and normal cells are the reasons for the selective higher cytotoxicity of antimicrobial peptides in cancer cells than normal cells, especially for the peptides with a positive charge [36]. The interaction between the antimicrobial peptides and the cell membranes may elevate the permeability of cancer cell membrane or even disrupt the plasma membrane. This greater diffusion promotes entry of anticancer drugs into the drug-resistant cancer cells [39]. Some studies have indicated that the antimicrobial peptides also have antimigratory effect, which could significantly prevent the migration of cancer cells [40]. The present study investigated the anticancer effect and the possible MDR reversal effects of some antimicrobial peptides.

First, the cytotoxicity assay on three antimicrobial peptides demonstrated that these peptides could kill drug-resistant cancer cells at a similar concentration compared with their IC_50_ values against the parental cells. This result may be beneficial for the future use of cancer therapy to overcome MDR caused by overexpression of ABCB1 and ABCG2. This result indicated that the peptides could abort drug resistant effects mediated by ABCB1 and ABCG2, which suggests that they may have the potential to reverse ABCB1- and ABCG2-mediated MDR. Furthermore, the tests on combination with anticancer drugs that are ABCB1 or ABCG2 substrates, showed that peptide XH-14C potentiates the cytotoxicity of paclitaxel and doxorubicin in ABCB1-overexpressing cancer cells. To restrict the reversal factors to only the overexpression of ABCB1, ABCB1 gene-transfected cell line HEK293/ABCB1 was used for further confirmation. In contrast, XH-14C had no effect on the cytotoxicity of cisplatin, a non-substrate drug of ABCB1 transporter, in all tested cell lines, indicating that XH-14C only affects ABCB1 transporter. The cytotoxicity of XH-14C and the drug resistance profile of ABCB1 substrates collectively demonstrated that XH-14C could act as a potential inhibitor of ABCB1.

Interestingly, the results of ATPase assay showed that XH-14C stimulated the ATPase activity of ABCB1. As we focus on the reversal agent of ABC transporters, to further examine the possible reversal effect of XH-14C on ABCB1 overexpressing cells, the interplay between XH-14C with ABCB1 was simulated by molecular docking analysis. The simulation results demonstrated that XH-14C could bind tightly to the drug-binding pocket of ABCB1 with several hydrogen bonds, which implied that XH-14C may compete with ABCB1 substrates as it could bind to ABCB1 much more tightly than the ABCB1 substrates. Such binding reduced the amount of the anticancer drugs being pumped out by ABCB1 transporter. In previous studies, a large proportion of tyrosine kinase inhibitors (TKIs), a kind of inhibitor involved in ABC transportermediated MDR reversal, could stimulate ATP hydrolysis [41]. Some are competitive inhibitors, as they could bind to the membrane transporter much more tightly than the substrate, preventing the substrate from being transported out by these transporters. With this assumption, the cytotoxicity assay of XH-14C should display some differences between the IC_50_ values of ABCB1-overexpressing cells to their parental cells as they might be substrates of ABCB1, which is conflicted with previous results that the cytotoxicity values of XH-14C were similar in ABCB1 overexpression cells compared to their corresponding parental cells. Therefore, XH-14C may not be an ABCB1 substrate but can stimulate ATPase activity by another mechanism. One of the third generation ABCB1 reversing TKI, tariquidar, is a non-competitive inhibitor able to stimulate ABCB1 ATPase activity. One possible assumption of this condition is that this kind of reversal agents could trap ABCB1 transporter in allosteric conformation which could stimulate the activity of ATPase while they are unable to be subjected to further conformational changes essential for drug outflow [42]. Although we did not have enough evidence to confirm this assumption, we speculate that XH-14C may actively stimulate the ATPase activity by capturing the ABCB1 transporter to a conformation that could not bind to ABCB1 substrates. The results of the docking analysis (Figure S3) showed that XH-14C and paclitaxel were binding to the same drug-binding pocket but different specific positions and interactions with different residues of ABCB1. Although the mechanisms of anticancer and reversal effect of these peptides are still not elucidated, according to the results here, these peptides show no substrate properties of ABC transporters. We mentioned in the introduction before that one possible anti-cancer mechanism of antimicrobial peptides is after binding to the cell membrane, by electrostatic attraction, antimicrobial peptides can disrupt membrane integrity, leading to the leakage and depolarization of metabolites. Another interpretation is a non-membrane mechanism that antimicrobial peptides target cell apoptosis after being transported inside the cell via membrane perturbation or formation of pores [32,33].Based on previous studies that investigated the anticancer effect of AMPs, these peptide samples might act on the mitochondria and stimulate the release of cytochrome C [40], which should also be assessed by evaluating cell apoptosis and intracellular glutathione change induced by the peptides using flow cytometry in the future.

As the ABCB1 transporter functions as an anticancer drug pump to enhance the efflux of anticancer drugs, the reversal agent should basically inhibit the function of the ABCB1 transporter. Drug accumulation and efflux assays using [^3^H]-paclitaxel, a substrate of ABCB1, showed that the intracellular level of [^3^H]-paclitaxel in ABCB1-overexpressing KB-C2 cells was dramatically enhanced in the presence of XH-14C compared with the control group. Although XH-14C did not completely elevate the intracellular concentration of [^3^H]-paclitaxel to a level of that in the parental cell line KB-3-1, its effects in augmenting drug accumulation were comparable to the positive reversal agent of ABCB1, verapamil.In this limited 4 h treatment with XH-14C, the results were consistent with the drug resistance profile of paclitaxel that the IC_50_ value in KB-C2 decreased as a result of the increased intracellular concentration of paclitaxel. Not only the accumulation assay, but also the time course of efflux assay of [^3^H]-paclitaxel verified that 3 μMof XH-14C could notably inhibit the efflux function of ABCB1 in KB-C2 cells, is more effective than verapamil, and almost similar to that in the parental KB-3-1 cells. One possible mechanism behind the block of the function of ABCB1 could be that XH-14C may inhibit the overexpression of ABCB1 in the MDR cells [3] so that the drug-resistant cells were re-sensitized to anticancer drugs. While the results of Western blotting showed that XH-14C did not downregulate the overexpressionof ABCB1 in KB-C2 cells with XH-14C treatment. Another possible reversal mechanism is that altering the localization of the ABCB1 transporter [3]. The results of immunofluorescence assay suggested that XH-14C did not affect the translocation of ABCB1 up to 72 h. There was no effect on either protein expression level or transporter localization even at higher concentration and longer time period. All the results above gave the conclusion that 3 μM XH-14C increased the accumulation of chemotherapeutic drugs by directly inhibiting ABCB1 transporter’s efflux function.

## 4. Materials and Methods

### 4.1. Chemicals and Reagents

All peptides were synthesized by solid phase methods using Fmoc N-terminal protected amino acids as previously described [43]. Before the in vitro study, all peptides were purified to >95% by RP-HPLC and the quality was analyzed and evaluated by mass spectrometry (MS) analysis. The phosphate buffered saline (PBS), paclitaxel, cisplatin, and topotecan were purchased from Sigma Chemical Co (St. Louis, MO, USA). Mitoxantrone was a product from Enzo life Sciences (Farmingdale, NY, USA).

### 4.2. Cell Lines and Cell Culture

The drug-selected ABCG2-overexpressing non-small cell lung cancer (NSCLC) cell line NCI-H460/MX20 and its parental NCI-H460 cell line, and the ABCB1 gene-transfected HEK293 cells, HEK293/ABCB1, and its parental empty vector transfected HEK293 cells, HEK293/pcDNA3.1 were provided by Drs. Susan E. Bates and Robert Robey (NIH, MD). The drug-resistant ABCB1 overexpressing human epidermal carcinoma cell line KB-C2 and its parental KB-3-1 cell line were generously provided by Dr. Shin-Ichi Akiyama (Kagoshima University, Kagoshima, Japan). All cell lines were cultured in complete Dulbecco’s modified Eagle’s medium(DMEM) (Hyclone, GE Healthcare Life Sciences, Pittsburgh, PA, USA) with the addition of 10% fetal bovine serum (FBS) and 1% penicillin/streptomycin (Hyclone, GE Healthcare Life Sciences, Pittsburgh, PA, USA). The transfected cell lines were selected in complete medium with addition of 2 mg/mL of Geneticin (Enzo Life Sciences, Farmingdale, NY, USA) [44] for selection, KB-C2 cells were continually treated with complete medium and 2 mg/mL of colchicine [45], and NCI-H460/MX20 cells were cultured with 20 ng/mL mitoxantrone added to the complete medium. All drug-treated cell lines were recovered for more than 2 weeks without treatment before their use. The culture condition was in a humidified incubator at 37 °C with 5% CO_2_.

### 4.3. MTT Colorimetric Assay

Cytotoxicity of the peptides on different cell lines and the cytotoxicity of ABCB1 and ABCG2 substrates were measured and calculated from the results of MTT assay [46] as previously described. The final absorbance of each well was measured by Microplate Spectrophotometer (Fisher Sci., Fair Lawn, NJ, USA) at 570 nm. Verapamil (Sigma Chemical Co., St. Louis, MO, USA) or Ko143 (Enzo life Sciences, Farmingdale, NY, USA) was used as the positive reversal agent, respectively.

### 4.4. ATPase Assay

The vanadate-sensitive membrane vesicles ABCB1 and ABCG2 ATPase activity were determined as previously described [45,47], and the ABCB1- and ABCG2-overexpressing cell membranes were purchased from BD Biosciences (San Jose, CA, USA). The ATPase activities were calculated based on the measurement of inorganic phosphate (IP)detected using a spectrophotometer at 800 nm.

### 4.5. Molecular Docking Simulation

The initial conformations of peptide XH-14C were generated using online server PEP-FOLD3 [48]. PEP-FOLD3 predicts peptide structures via de novo approaches from amino acid sequencing based on structural alphabet SA letters [48]. The generated structures were then subjected to a 10 ns molecular dynamics equilibration using Desmond (DE Shaw Research Group, NY, USA) [49]. The simulation protocol was similar to that previously described, with modification [45]. In brief, the simulation system was first built in the Maestro Module System Builder. The NpT runs under 300 K and 1.015 bar pressure for 10 ns. The conformations of XH-14C were picked from time frames when the system reached dynamic equilibration. ABCB1 model (4M2T) was obtained from the Protein Data Bank (PDB). Peptide–protein docking was performed using rigid-body docking programs ZDOCK, which uses the Fast Fourier Transform based docking algorithms [50]. The best scoring pose was selected for further visualization and analysis. Ligand–protein interactions were analyzed using LigPlot+ [51].

### 4.6. Drug Accumulation and Efflux Assay

[^3^H]-paclitaxel (31 Ci/mmol, Moravek Biochemicals, Brea, CA, USA) was used to measure the drug accumulation and efflux as previously described [46]. The only difference is that after the cells were seeded evenly in 24-well plate, the cells were incubated in the presence or absence of XH-14C and a parallel positive reversal agent for 72 h. Liquid scintillation cocktail used to measure the radioactivity was purchased from MP Biomedicals, Inc (St. Ana, CA, USA) and the radioactivity of different groups were read with the Packard TRI-CARB1 190‘A liquid scintillation analyzer. A parallel well of cells for each group was set up for cell number counting at the end of the assay for the purpose of data normalization.

### 4.7. Western Blotting Analysis

To investigate whether XH-14C can alter the expression of ABCB1, Western blotting was performed as previously described [52] with the primary monoclonal anti-ABCB1 (MDR1) antibody produced in mouse (Sigma Chemical Co, St. Louis, MO, USA), GAPDH Mouse Anti-Human Clone: GA1R (Thermo Fisher Scientific Inc., Rockford, IL, USA), and the anti-mouse HRP-linked secondary antibody (Cell Signaling Technology, Danvers, MA, USA), all diluted to 1:1000. The developed protein expression level was measured and analyzed by ImageJ software (NIH, Bethesda, MD, USA).

### 4.8. Immunofluorescence Assay

To verify the subcellular localization of ABCB1, immunofluorescence assay was performed as previously described [53] with the primary monoclonal anti-ABCB1 (MDR1) antibody produced in mouse (Sigma Chemical Co, St. Louis, MO, USA) and Alexa Fluor 488 rabbit anti-mouse secondary antibody (Thermo Fisher Scientific Inc., Rockford, IL) both diluted to 1:1000. 4′,6-diamidino-2-phenylindole (DAPI) (Thermo Fisher Scientific Inc., Rockford, IL, USA) dissolved in 1 μg/mL solution with 1× PBS was used to counterstain the nuclei of the cancer cells.

### 4.9. Statistical Analysis

All the values were calculated and presented as mean ± SD. All experiments were performed at least three times independently. Statistical differences between multiple groups were calculated by one-way ANOVA followed by Dunnett’s test and were considered significant when p value was below 0.05.

## 5. Conclusions

This present study reports for the first time that a novel antimicrobial peptide could re-sensitize ABCB1 overexpression-mediated MDR cancer cells by directly inhibiting the efflux function of ABCB1 transporter. Past potent reversal agents investigated in vitrohad the problems of bioavailability, neurotoxicity, and synergetic effects, the much larger molecular peptides and their specific binding to cancer cells may avoid these possible disadvantages. This study identified a promising therapeutic strategy and may provide a potential clinical direction on overcoming ABCB1-mediated MDR in cancers. In the future, the clinical therapeutic effect of XH-14C needs to be explored in in vivo studies with xenograft animal models and clinical trials. Also, pharmacokinetic studies are necessary to verify the bioavailability of this peptide. Further studies are warranted to confirm the structure specialty of these peptides and whether they could be contributed to helping patients suffering chemotherapy-induced MDR and improving clinical outcomes in patients receiving chemotherapy.

## Figures and Tables

**Table 1:** The cytotoxicity of ABCB1 substrates with or without a reversal agent.

Treatment	IC_50_^1^ (nM) (RF ^2^)	
	KB-3-1	KB-C2
Paclitaxel	3.0 ± 0.5 (1.00)	1310 ± 271 (437)
+ XH-14A (3 μM)	4.0 ± 0.3 (1.33)	1767 ± 224 (589)
+ XH-14B (3 μM)	3.0 ± 0.9 (1.00)	441 ± 9.0 (147)
+ XH-14C (3 μM)	2.0 ± 0.6 (0.67)	28.0 ± 4.0 ^#^ (9.33)
+ Verapamil (3 μM)	2.0 ± 0.4 (0.67)	10.0 ± 6.0 ^#^ (0.33)
Doxorubicin	1276 ± 189 (1.00)	67600 ± 2459 (53.0)
+ XH-14A (3 μM)	1187 ± 174 (0.93)	61030 ± 5099 (47.8)
+ XH-14B (3 μM)	1010 ± 204 (0.79)	15010 ± 1310 (11.8)
+ XH-14C (3 μM)	955 ± 102 (0.75)	1594 ± 153 ^#^ (1.25)
+ Verapamil (3 μM)	1026 ± 136 (0.80)	832 ± 112 ^#^ (0.65)
Cisplatin	1635 ± 487 (1.00)	1764 ± 377 (1.08)
+ XH-14A (3 μM)	1626 ± 223 (0.99)	1610 ± 283 (0.98)
+ XH-14B (3 μM)	1672 ± 361 (1.02)	1650 ± 283 (1.01)
+ XH-14C (3 μM)	1690 ± 455 (1.03)	1795 ± 353 (1.10)
+ Verapamil (3 μM)	1685 ± 482 (1.03)	1796 ± 256 (1.10)

^1^ IC_50_ values were calculated from at least three independent experiments performed in triplicate and finally represented as mean ± SD with unit of nM.^2^ RF, resistant fold, which was calculated using the IC_50_ in the drug-selected ABCB1-overexpressing cancer cell line KB-C2 divided by the IC_50_ in the drug-sensitive cancer cell line KB-3-1. ^#^, represents *p <* 0.001, compared to the value of KB-C2 control group.

**Table 2:** The cytotoxicity of ABCB1 substrates with or without a reversal agent.

Treatment	IC_50_^1^ (nM) (RF ^2^)	
	HEK293/pcDNA3.1	HEK293/ABCB1
Paclitaxel	14.3 ± 1.53 (1.00)	305.8 ± 45.4 (21.38)
+ XH-14A (3 μM)	15.1 ± 3.14 (1.06)	240.4 ± 25.2 (16.85)
+ XH-14B (3 μM)	15.1 ± 2.45 (1.06)	209.2 ± 28.1 (14.63)
+ XH-14C (3 μM)	13.5 ± 2.65 (0.95)	16.5 ± 2.45 ^#^ (1.15)
+ Verapamil (3 μM)	14.7 ± 3.69 (1.03)	11.6 ± 2.59 ^#^ (0.82)
Doxorubicin	1226 ± 1.88 (1.00)	32646 ± 4807 (26.6)
+ XH-14A (3 μM)	1254 ± 172 (1.02)	23054 ± 2830 (18.8)
+ XH-14B (3 μM)	1176 ± 238 (0.96)	19423 ± 2303 (15.8)
+ XH-14C (3 μM)	1420 ± 147 (1.16)	1316 ± 357 ^#^ (1.07)
+ Verapamil (3 μM)	1271 ± 155 (1.04)	1288 ± 259 ^#^ (1.05)
Cisplatin	2275 ± 489 (1.00)	2595 ± 246 (1.14)
+ XH-14A (3 μM)	2268 ± 368 (1.00)	2476 ± 269 (1.09)
+ XH-14B (3 μM)	2272 ± 487 (1.00)	2358 ± 392 (1.04)
+ XH-14C (3 μM)	2365 ± 359 (1.04)	2559 ± 175 (1.12)
+ Verapamil (3 μM)	2296 ± 186 (1.01)	2185 ± 228 (0.96)

^1^ IC_50_ values are calculated from at least three-time independent experiments performed in triplicate and finally represented as mean ± SD with unit of nM.^2^ RF, resistant fold, which was calculated using the IC_50_ in the transfected ABCB1-overexpressing cell line HEK293/ABCB1 divided by the IC_50_ in the transfected empty-vector cell line HEK293/pcDNA3.1. ^#^, represents *p <* 0.001, compared to the value of HEK293/ABCB1 control group.

## Supplementary Materials

The following are available online at https://www.mdpi.com/2072-6694/12/7/1963/s1, Figure S1: Dose-response curve of **A**) XH-14A, **B**) XH-14B, and **C**) XH-14C on drug-selected ABCG2-overexpressing cell lines (NCI- H460/MX20) and its parental drug-sensitive cell line (NCI-H460) with gradient concentrations, Figure S2: The vanadate sensitive ABCG2 transporter specific ATPase activity does not change by XH-14C, Figure S3: The whole blot of Western blotting results, Table S1: The cytotoxicity of ABCG2 substrates with or without combination of a reversal agent.
