# Supplementary material for: The Multidrug Resistance-Reversing Activity of a Novel Antimicrobial Peptide
Source: Cancers (Basel). 2020 Jul 19;12(7):1963. doi: 10.3390/cancers12071963 (PMC7409168; doi:10.3390/cancers12071963)
Supplement: Supplementary file 1 [file cancers-12-01963-s001.pdf]

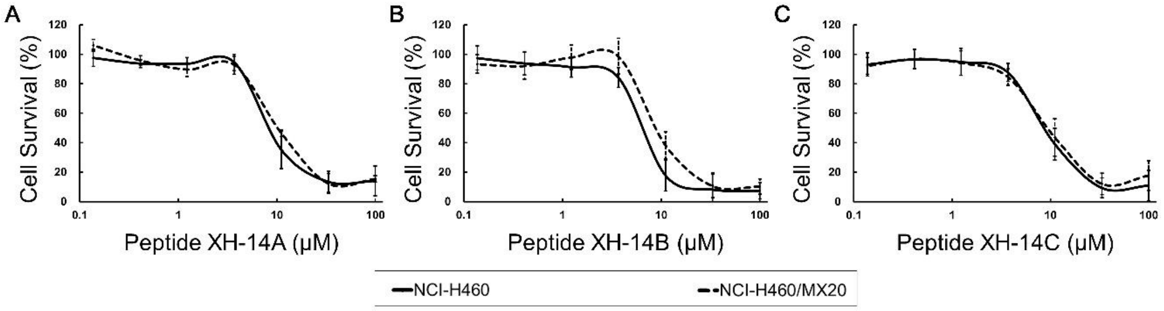

Figure S1. Dose-response curve of A) XH-14A, B) XH-14B and C) XH-14C on drug-selected ABCG2-overexpressing cell lines (NCI-H460/MX20) and its parental drug-sensitive cell line (NCI-H460) with gradient concentrations. Each point with error bar represents the mean ± SD of the cytotoxicity with different concentrations calculated from at least three independent experiments performed in triplicate.

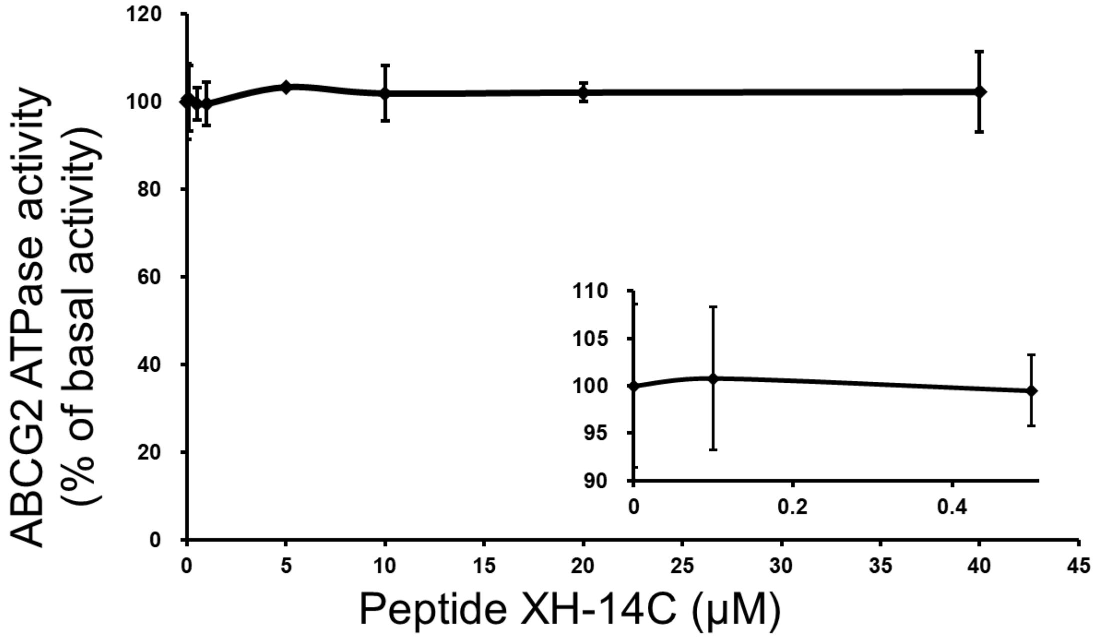

Figure S2. The vanadate sensitive ABCG2 transporter specific ATPase activity does not change by XH-14C. Gradient concentration of XH-14C (0-40 µM) as x-axis and ABCG2 ATPase activity represented in percentage of basal activity as y-axis was plotted. The small inner figure plotted the lower concentrations (0-0.5 µM) of XH-14C versus ATPase activity. The points with error bar represent the mean ± SD calculated from three independent experiments.

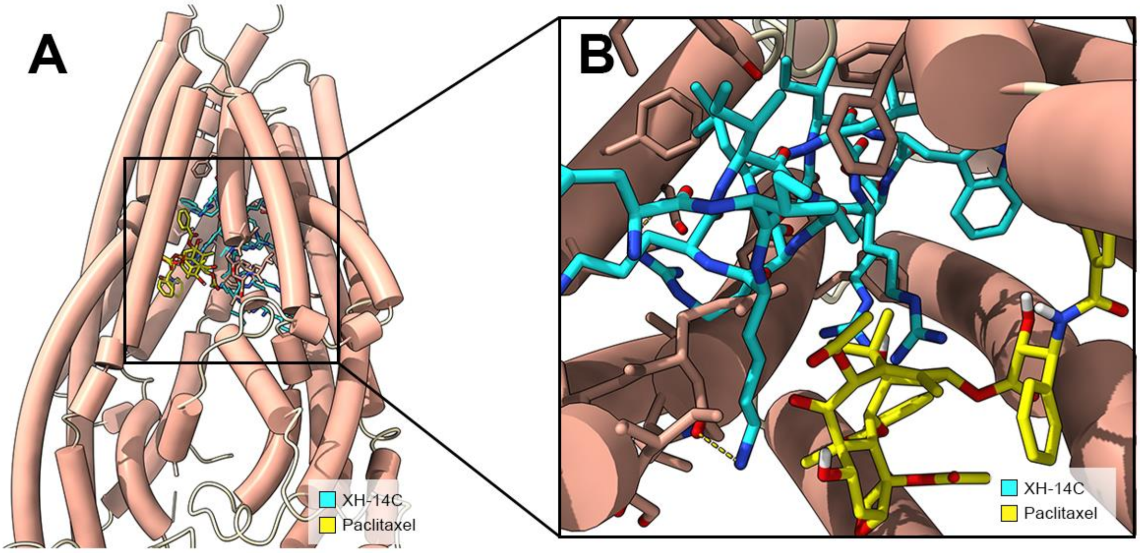

Figure S3. Docking simulation of XH-14C and paclitaxel with ABCB1. A. Best scoring poses of XH-14C and paclitaxel in the drug binding pocket of ABCB1. XH-14C was depicted as cyan sticks and paclitaxel was depicted as yellow sticks. ABCB1 (4M2T) was depicted as colored tubes. B. Details of the positions of XH-14C and paclitaxel with ABCB1 binding pocket.

Table S1. The cytotoxicity of ABCG2 substrates with or without combination of a reversal agent.

Treatment	IC_{50}[1] (μM) (RF[2])	
	NCI-H460	NCI-H460/MX20
Mitoxantrone	0.039 ± 0.005 (1.00)	36.55 ± 4.53 (937)
+ XH-14A (3 μM)	0.055 ± 0.003 (1.41)	34.28 ± 4.13 (879)
+ XH-14B (3 μM)	0.051 ± 0.008 (1.31)	38.49 ± 3.55 (987)
+ XH-14C (3 μM)	0.041 ± 0.006 (1.05)	36.85 ± 3.95 (945)
+ Ko143 (3 μM)	0.043 ± 0.004 (1.10)	0.089 ± 0.0040[#] (2.28)
Topotecan	0.049 ± 0.006 (1.00)	39.16 ± 4.85 (799)
+ XH-14A (3 μM)	0.062 ± 0.004 (1.27)	34.95 ± 4.21 (713)
+ XH-14B (3 μM)	0.040 ± 0.009 (0.82)	35.96 ± 4.04 (734)
+ XH-14C (3 μM)	0.042 ± 0.005 (0.86)	37.83 ± 3.16 (772)
+ Ko143 (3 μM)	0.037 ± 0.003 (0.76)	0.096 ± 0.0064[#] (1.96)
Cisplatin	2.76 ± 0.23 (1.00)	3.15 ± 0.47 (1.14)
+ XH-14A (3 μM)	3.17 ± 0.31 (1.15)	3.29 ± 0.26 (1.19)
+ XH-14B (3 μM)	2.82 ± 0.22 (1.02)	2.96 ± 0.31 (1.07)
+ XH-14C (3 μM)	2.77 ± 0.32 (1.00)	3.17 ± 0.33 (1.15)
+ Ko143 (3 μM)	3.47 ± 0.35 (1.26)	3.40 ± 0.46 (1.23)

[1] IC_{50} values are calculated from at least three independent experiments performed in triplicate and finally represented as mean ± SD with unit of nM. [2] RF, resistant fold, calculated by the IC_{50} in the drug-selected ABCG2-overexpressing cancer cell line NCI-H460/MX20 divided by the IC_{50} in the drug-sensitive cancer cell lineNCI-H460. [#], represents $p < 0.001$, compared to the value of NCI-H460/MX20 control group.
